# Prediction of Suicide-Related Events by Analyzing Electronic Medical Records from PTSD Patients with Bipolar Disorder

**DOI:** 10.3390/brainsci10110784

**Published:** 2020-10-27

**Authors:** Peihao Fan, Xiaojiang Guo, Xiguang Qi, Mallika Matharu, Ravi Patel, Dara Sakolsky, Levent Kirisci, Jonathan C. Silverstein, Lirong Wang

**Affiliations:** 1 Department of Pharmaceutical Sciences, Computational Chemical Genomics Screening Center, University of Pittsburgh School of Pharmacy, Pittsburgh, PA 15206, USA; pef14@pitt.edu (P.F.); xig53@pitt.edu (X.G.); xiq24@pitt.edu (X.Q.); 2 Department of Statistics and Department of Economics, University of Pittsburgh School of Arts & Sciences, Pittsburgh, PA 15213, USA; mam802@pitt.edu; 3 Department of Pharmacy and Therapeutics, University of Pittsburgh School of Pharmacy, Pittsburgh, PA 15213, USA; RaviPatel@pitt.edu; 4 Department of Psychiatry, University of Pittsburgh School of Medicine, Pittsburgh, PA 15213, USA; sakolskydj@upmc.edu; 5 Department of Pharmaceutical Sciences, University of Pittsburgh School of Pharmacy, Pittsburgh, PA 15213, USA; 6 Department of Biomedical Informatics, University of Pittsburgh School of Medicine, Pittsburgh, PA 15213, USA

**Keywords:** PTSD, bipolar disorder, machine learning, random forest, suicide-related events, model decomposition

## Abstract

Around 800,000 people worldwide die from suicide every year and it’s the 10th leading cause of death in the US. It is of great value to build a mathematic model that can accurately predict suicide especially in high-risk populations. Several different ML-based models were trained and evaluated using features obtained from electronic medical records (EMRs). The contribution of each feature was calculated to determine how it impacted the model predictions. The best-performing model was selected for analysis and decomposition. Random forest showed the best performance with true positive rates (TPR) and positive predictive values (PPV) of greater than 80%. The use of Sertraline, Fentanyl, Aripiprazole, Lamotrigine, and Tramadol were strong indicators for no SREs within one year. The use of Haloperidol, Trazodone and Citalopram, a diagnosis of autistic disorder, schizophrenic disorder, or substance use disorder at the time of a diagnosis of both PTSD and bipolar disorder, predicted the onset of SREs within one year. Additional features with potential protective or hazardous effects for SREs were identified by the model. We constructed an ML-based model that was successful in identifying patients in a subpopulation at high-risk for SREs within a year of diagnosis of both PTSD and bipolar disorder. The model also provides feature decompositions to guide mechanism studies. The validation of this model with additional EMR datasets will be of great value in resource allocation and clinical decision making.

## 1. Introduction

Approximately 800,000 people worldwide die from suicide every year [1]. Suicide is the 10th leading cause of death in the United States, with 48,000 deaths occurring in 2018 [2]. Because the rate of suicide among all deaths has continued to increase since 1999 [2,3,4,5,6,7,8], reversing the suicide rate has been prioritized by the World Health Organization [1].

Multiple studies have identified factors related to suicide. These factors include age, gender, and alcohol abuse [9,10]. However, it is hard to quantify the influence of these factors using traditional statistical methods [11,12,13]. With the rise of machine-learning (ML) algorithms, several successful studies have predicted suicide risk based on different ML-based methods with good accuracy [14,15,16,17].

However, few studies have focused on risk factors among high-risk populations that may require more intensive interventions to prevent suicides. Some research suggest that, among all mental disorders, bipolar disorder contributes most to the risk for suicide [18]. This risk can be higher when comorbidity exists with other psychiatric disorders [19]. Patients with bipolar disorder alternate between manic and depressive episodes [20], leading to considerable impairment of life quality [21,22]. Approximately 1% of the worldwide population suffer from bipolar disorder [23,24].

Although considered a controversial suicide risk factor, post-traumatic stress disorder (PTSD) is one of the most common comorbidities of bipolar disorder [25,26,27], with a lifetime comorbidity rate of 16%–39% [28]. PTSD is a trauma- and stressor-related disorder with the major post-trauma symptoms being fear-based re-experiencing, anhedonia, dysphoric mood stages, or dissociative symptoms [29]. The lifetime prevalence of PTSD is 7.8% [30]. Among patients with bipolar disorder, the time spent with illness as well as SREs (ideation, attempts, and deaths) are significantly higher in patients also diagnosed with PTSD [27,31,32].

Suicide prevention strategies have been proven to substantially decrease the suicide rate [33,34,35,36]. The importance of recognizing depression and suicidal tendencies has been emphasized in several reviews [33,35,37,38]. However, comprehensive suicide prevention programs consume time, labor, and resources that limit the application of care to all patients with bipolar disorder and PTSD. A time-, labor-, and resource-saving quantitative measurement for SREs in this high-risk population is needed to guide clinical decision-making and to help distribute resources to the patients who can most benefit from them.

An ML-based random forest model was constructed to identify and quantify risk factors that induce suicide among patients diagnosed with both bipolar disorder and PTSD. Using factors extracted from electronic medical records (EMRs) of patients with both diagnosis of bipolar disorder and PTSD, patients with a higher risk of an SRE within a year were identified. The model focused primarily on predictors like baseline disease conditions or pharmacy records which can be easily obtained and are less likely to be biased by subjective factors. Extra emphasis was placed on lithium usage, as previous studies have demonstrated that lithium was effective in preventing death by suicide in patients with mental disorders [34,39,40].

## 2. Materials and Methods

### 2.1. Data Sources

Data was collected from the EMRs of patients seen at UPMC (University of Pittsburgh Medical Center) facilities between 2004 and 2019. The cohort of patients with PTSD and bipolar disorders were identified based on the International Classification of Diseases (ICD) 9 and 10 codes for these disorders. Records for these patients, including demographics, medication usage, encounters and diagnosis of comorbid diseases, were extracted from the EMR systems as an IRB (Institutional Review Board)-approved HIPAA (Health Insurance Portability and Accountability Act) Limited Data Set. These data included the dates for each transaction, using the University of Pittsburgh’s research data warehouse. After data extraction, a sub-population that was diagnosed with both PTSD and bipolar disorder was created.

SREs for this sub-population were identified from the EMRs. The diagnosis codes included ICD9 and ICD10 for suicidal ideation, attempt, and death based on literature reports [3,41]. The diagnosis table was searched using the keywords ‘suicide’, ‘suicidal’ and ‘intentional self-harm’. Events of undetermined intent (Y10–Y34) were not considered. The lack of well-defined codes like X60–X70 in the list might indicate a bias of coding preference in the UPMC EMR system. 

The dates of first PTSD and bipolar disorder diagnoses of patients were extracted, with the later date assigned to each patient as Both Diagnosed Time (BDT). SRE predictions were made at this time point. Patients’ ages when they were diagnosed with both disorders were determined. Patients were excluded that had SREs before BDT since the SREs may have not be casually linked to the diagnosis of both PTSD and bipolar disorder. The time interval between BDT and an SRE was calculated for high-risk patients. SREs within one year after BDT were marked as 1 (event identified). Patients with follow-up times beyond one year that did not have an SRE or had it more than a year after BDT, were marked as 0 (event not identified). Comorbid medical disorders were also documented and categorized into 12 disease categories that used only ICD9 codes [17]. These ICD9 codes were mapped to ICD10 codes using the service provided at http://www.icd10codesearch.com/. Patients having dipolar disorder and PSTD diagnosis codes within a year prior to BDT were considered as having these comorbid diseases.

Three major classes of medications taken into consideration as predictors were mood stabilizers, antipsychotics, and antidepressives, and were extracted from DrugBank [42] (https://www.drugbank.ca/). Patients were marked as taking these medications if they had been prescribed within one year prior to their BDT to find predictor information for SREs at the point of BDT. Included in this study were 75 extracted medications from the three classes that were matched with medications recorded in the EMR system. Medications included in this study are: Almotriptan, Amitriptyline, Amoxapine, Amphetamine, Aripiprazole, Asenapine, Brexpiprazole, Bupropion, Buspirone, Carbamazepine, Cariprazine, Chlorpheniramine, Chlorpromazine, Citalopram, Clomipramine, Clozapine, Desipramine, Desvenlafaxine, Dexmethylphenidate, Dextromethorphan, Dihydroergotamine, Doxepin, Duloxetine, Eletriptan, Escitalopram, Fentanyl, Flibanserin, Fluoxetine, Fluphenazine, Fluvoxamine, Frovatriptan, Haloperidol, Iloperidone, Imipramine, Lamotrigine, Levomilnacipran, Lithium, Loxapine, Lurasidone, Maprotiline, Meperidine, Methadone, Milnacipran, Mirtazapine, Naratriptan, Nefazodone, Nortriptyline, Olanzapine, Paliperidone, Paroxetine, Perphenazine, Phenelzine, Pimozide, Promethazine, Protriptyline, Quetiapine, Rasagiline, Risperidone, Rizatriptan, Ropinirole, Rotigotine, Selegiline, Sertraline, Sumatriptan, Tapentadol, Thiothixene, Tramadol, Tranylcypromine, Trazodone, Trifluoperazine, Venlafaxine, Vilazodone, Vortioxetine, Ziprasidone, and Zolmitriptan.

### 2.2. Software and Model Setup

The analysis algorithm was written in the Python programming language in a Jupyter notebook [43]. The ML-based models and calibration curves were developed by using scikit-learn 0.20.0 [44]. The key Python libraries used in this analysis were SciPy [45], NumPy [46] and Pandas [47]. 

Several different ML-based classifiers were tested, including logistic regression [48], random forest [49], decision tree [50], K-nearest neighbors [51], Naïve Bayes [52] and support vector machine [53]. All models were set at a random state of 42 to ensure reproducibility while the other hyper-parameters were left at default settings. The random state seeded the random number generator used in the models. For the final random forest model, we set estimators to 100 and the maximum number of features to the square root of the number of features.

ML-based models frequently encounter datasets that are heavily imbalanced—the number of samples in the different classes are distributed unevenly—which affects their learning phases and subsequent predictions. An over-sampling procedure based on the Synthetic Minority Oversampling Technique (SMOTE) [54] was performed prior to conducting the analysis. The over-sampling procedure creates new samples by connecting inliers and outliers from the original dataset [54]. The resampled dataset was split into training and test datasets randomly in a 4:1 ratio. Only the training set was oversampled with SMOTE so that the test set contained the original subjects in the dataset.

Many socioeconomic factors have been reported to play important roles in suicide prediction [55]. However, data from only the EMR were used as the predictors, variables, or features for modeling: (a) demographic data, including gender and age at BDT; (b) number of emergency department (ED) visits and diagnoses within one year prior to the BDT; (c) medication usage within one year prior to the BDT, including medication orders, dispenses, and fills. Medication usage data was coded by whether patients had taken these medications within one year prior to their BDT.

Predictor or variable importance was calculated to assess key factors in SRE prediction. In the random forest algorithm, predictor importance was quantified by evaluating the decrease in “node impurity” at each split across all decision trees in the forest [56]. In the simplest case, node impurity can be considered as the difference in measurement from controls at a node. The random forest module uses these measures to estimate variances in nodes across trees. The nodes with maximized response variances are those that have greater contributions to the differences in categories of cases and have a greater impact on the model’s ability to predict outcomes.

Since patients with SREs are a minor class in our dataset, model performance was based on true positive rate (TPR), positive predictive value (PPV), and negative predictive value (NPV) calculated as follows (Equation (1)):

(1) $$\mathrm{TPR}=\frac{\mathrm{True}\;\mathrm{Positives}}{\left(\mathrm{True}\;\mathrm{Positives}+\mathrm{False}\;\mathrm{Negatives}\right)}\;\mathrm{PPV}=\frac{\mathrm{True}\;\mathrm{Positives}}{\left(\mathrm{True}\;\mathrm{Positives}+\mathrm{False}\;\mathrm{Positives}\right)}\;\mathrm{NPV}=\frac{\mathrm{True}\;\mathrm{Negatives}}{\left(\mathrm{True}\;\mathrm{Negatives}+\mathrm{False}\;\mathrm{Negatives}\right)}$$

Random forest results were interpreted using the python package TreeInterpreter 0.2.2 (https://github.com/andosa/treeinterpreter), which allowed the (a) decomposition of each prediction into feature contribution components in the training set mean and (b) identification of those features that affect the difference and their contribution. In the model, all features will make contributions to the predication about an instance whether positive or negative. If the value of a feature’s contribution was positive (SRE), the prediction value was scored as 1. If the feature’s contribution was negative (no SRE), the prediction value was scored as 0.

## 3. Results

### 3.1. Model Construction and Performance

**Figure 1:**
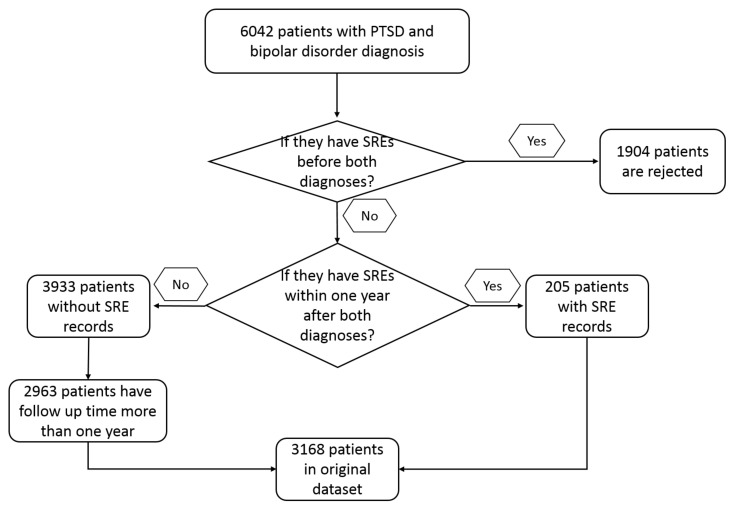
Inclusion process of patients with PTSD and bipolar disorder.

A total of 6042 patients with PTSD and bipolar disorder were identified from the EMR system by ICD9 and ICD10 codes (Appendix A). Of this population, 4138 of them had no records of SRE before BDT. Among these 4138 patients, 205 were identified as having SREs within one year after BDT, while 3933 of them did not have SREs in the same time period. Patients with follow up time less than one year and no reported SRE (970) were excluded from this study. The filtered 2963 subjects were oversampled into a balanced dataset by SMOTE as described above. After data resample and split, the training dataset contained 4726 subjects with 2363 subjects marked as 1 and 2363 subjects marked as 0. The inclusion process is described in Figure 1 and the baseline patient characteristics are shown in Table 1. Significant differences among patients with and without SREs because of gender, age, and ED visits may be contributing variables in this study.

ML-based models were trained and evaluated with the data generated by the resample procedures. Performances of all the models are shown as the means from a 5-fold stratified cross-validation process (Table 2). TPR and PPV were prioritized since the model should be able to identify the high-risk population within the precision constraints relevant to the data. Random forest was superior at retrieving positive cases with less false positives with an exceptional high PPV (Table 2). Random forest achieved an accuracy of 92.4%, an area under curve (AUC) of 95.6%, an F1 score of 0.879, and an area under receiver operating characteristic (ROC) curve of 0.820. The random forest model was chosen as the predictive model in the following analysis.

### 3.2. Model Decomposition and Feature Importance Analysis

A decomposition analysis on the decision trees generated by the random forest algorithm was conducted to better understand the contributions of each factor on SRE predictions. All features in the model were examined individually to determine if the feature provided positive contributions. Such an approach allowed a minimization of the data volume needed to make an accurate prediction and to reduce computation expenses.

Ninety-two features were used in the model including disease categories 1–12, the seventy-five medications mentioned above, age, gender and ED visits. Among them, only age and ED visits were continuous variables, and all other features were categorical. In order to find the features that are necessary for the model and to minimize the data requirement, feature importance was calculated using the method implemented in the package. Feature importance was calculated as the decrease in node impurity weighted by the probability of reaching that node. The node probability was calculated by the number of samples that reach the node, divided by the total number of samples. The higher the value the more important the feature [57]

**Figure 2:**
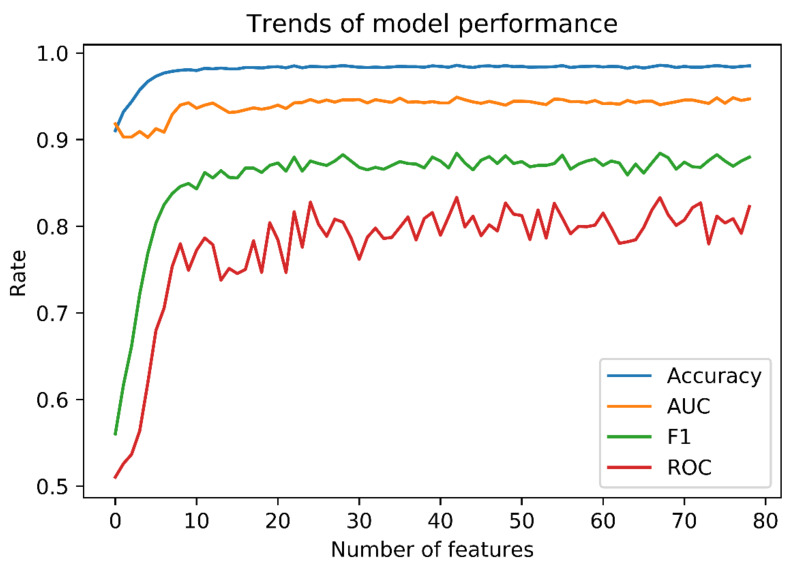
Trends of model performances using different number of features. As shown in the figure, the performance of model improved as we included more features in the model and it reached a plateau at around 30 features. Therefore, we were able to achieve the similar model performance with much less features.

Multiple random forest tests, which included the top most important features, were performed to retrain the model and test its performance. The performance of model improved as the number of features with high importance increased (Figure 2). The performance curves reached a plateau at approximately 30 features, then maintained a performance similar to the original model we trained using all 90 features. As a result, the 30 most important features (Table 3) were used to train a simplified random forest model.

**Figure 3:**
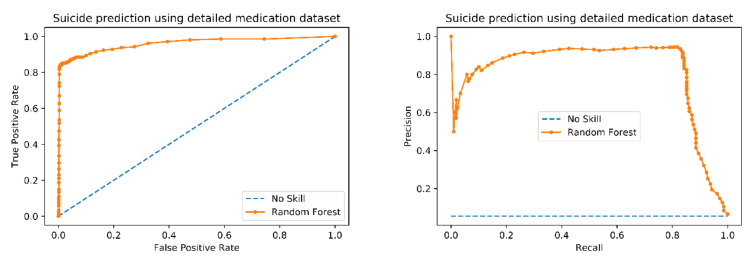
ROC curve and Precision-Recall Curve for modified model. These two curves are the most common measures to demonstrate the performance of a prediction model. Our model showed a F1 score of 0.877 and the area under ROC is 0.809 indicating a good precision and recall performance. This means our model is very accurate and has a high sensitivity and specificity.

A simplified random forest model was built using the top 30 features. The ROC and the Precision-Recall graphs of the new model were plotted (Figure 3). The random forest model outperforms the no skill (random) model in both graphs (Figure 3). The simplified model yielded an accuracy of 98.3%, an AUC of 95.9% (similar to the original model performance), an F1 score of 0.868, and an ROC of 0.811. The performance parameters for the retrained model achieved a high TPR and PPV with the 30 selected features, again similar to the original model performance (Table 2; Table 4). These results indicate that the random forest model is sensitive to patients who had SREs and can predict SREs correctly. Every feature that impacted the final prediction using random forest was processed through the decomposition algorithms from treeinterpreter. 

The random forest model was used to predict how each feature could impact the possibility of having a SRE within one year after being BDT on all 3168 patients in the dataset. Of the 3168 patients, the model correctly predicted SREs from 3120 of them. Contribution values (negative and positive) of the features to correctly predicted presence of SREs within 1 year were calculated.

**Figure 4:**
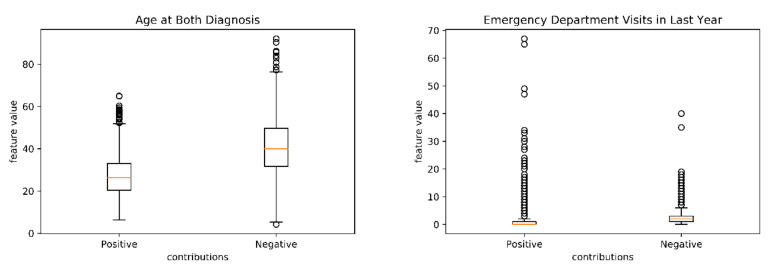
Distribution of age and ED visits in correctly predicted cases. Age distributions and ED visits are significantly different in two groups. Younger patients and patients with more ED visits are associated with higher-risk of SREs.

The distributions for two continuous datasets, age and ED visits were investigated (Figure 4). The age and ED distributions between positive and negative scores were significantly different (*p* < 0.001) (Figure 4). Younger ages and more ED visits are associated with a higher risk of having SREs.

**Figure 5:**
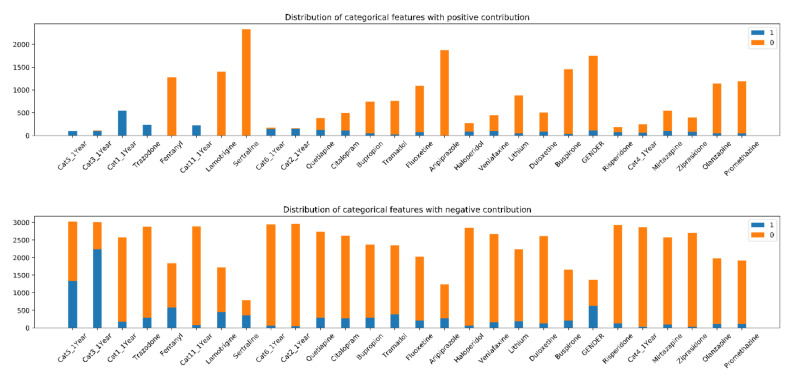
Distribution of feature values with positive and negative contributions. Most 0 values are associated with a higher risk of suicide and 1 are considered having lower risks. 0 means that the patients did not have the disease or did not take the medication and 1 means they did. Some features showed obvious separation in contributions by values which means the values of these features are strongly associated with the final prediction( CatN_1Year: Disease Category N in last year (N = 1, 2, 3, 4, 5, 6 and 11)).

The distribution of the 28 categorical features provided an insight into how the individual features impacted the SREs of individual cases (Figure 5). Generally speaking, value 1 tended to make a positive contribution compared to 0 across all features. Specifically, features such as Fentanyl, Aripiprazole, Disease category 11, Disease category 2 and Disease Category 6 showed obvious associations between contributing groups and feature values. The value distributions of features are different in positive and negative contributing groups (Figure 4) and these shifts can provide information about the impact a feature may have on SREs. The difference in value distributions of features were examined using a chi-square test (Table 5) and as a percentage in positive and negative contributing groups. If a feature has no or little association with the final prediction, the percentages of patients taken medication or have the comorbid disease in positive and negative contributing groups should be similar to the percentage of 1 in the whole population. If the percentage of patients taken medication or have the comorbid disease in positive or negative contributing group significantly differs from that of the whole population and each other, it suggests a possible mechanistic association between this feature and the potential risk for an SRE. For example, 11.6% of the participants have taken Sertraline. They account for 0% of the positive contributing groups and 45.9% of negative contributing groups. It can be concluded that taking Sertraline is predictive for no SREs within one year. High-importance features with an obvious separation pattern among the population groups have also been identified (Table 3). This indicates that the values of these features can greatly impact the final SRE predictions and may inform future mechanism studies.

All features except Olanzapine showed a significant difference between their distributions in positive and negative contributing groups. This is the result we are expecting because all the features in Figure 5 have been selected through the drop column feature importance test and were identified as important for the model to make the prediction. If the value of a certain feature does not provide significant differences in the percentages among the groups, it is likely that it has no benefit in for predicting SREs and will be dropped in the previous step. The results shown in Table 5 provided additional support to our feature selection process above.

## 4. Discussion and Conclusions

Prior studies have found that the ML-based methods perform better at identifying suicide risks in large populations of patients than traditional methods. The accuracies of these studies are reported to be between 0.76–0.79 with AUCs generally between 0.80–0.90 [15,17,58]. The objective of this study was to find an ML-based method that identifies patients at high-risk for SREs—patients diagnosed with both PTSD and bipolar disorder. This study demonstrated an accuracy of 0.92 and an AUC of 0.956 using the random forest method. The random forest model can accurately predict those patients at higher risk of SREs as evaluated with TPR and PPV tests. Sub-populations suffering from certain disorders and taking certain medications can be distinguished from a larger population as having a higher risk for SREs.

Different features have different contributions to the prediction of SREs. These features are mental disorders and drug administration history within one year. As discussed by Sanderson et al. [17], mental health diagnoses were separated into twelve disease categories based on their ICD9 codes (Appendix B). Patients suffering from comorbid diseases at BDT are more likely to have SRE within a year. These comorbid diseases include: Category 11, autistic disorder-current and disturbance of conduct; Category 3, mood disorders and adjustment disorders; Category 4, other psychotic disorders; and Category 5, acute stress reactions. Several studies have reported results that diseases in Category 11 are more likely to trigger SRE [59,60,61,62]. Numerous studies have provided evidence that mental disorders have the potential to increase the risk of SREs [63,64]. This evidence is supported by the results of the random forest model presented here. 

For the unselected features, it does not mean that these features may not be useful predictors of SREs. The aim of the feature selection process to use a sufficient but minimal number of features for the model to achieve optimal prediction results. It was found that optimal results were found using 30 features and that the addition of additional features did not affect the results. The distribution of all categorical features is attached (Appendix C). The impurity-based feature importance can be misleading for high cardinality features and continuous variables (age and ED visits) [65]. For this reason, the distribution of these two variables were examined first to ensure that their association with SREs are not the result of biased algorithms. 

With medication usage as the feature, some of them showed a much higher proportion in the negative contributing group compared to either the whole population or the positive contributing groups (Fentanyl, Levomilnacipran, Sertraline, Aripiprazole, Tramadol, Lamotrigine, Sertraline, and Fluoxetine). These medications are considered to reduce the risk of SREs within one year in our model. Other investigators have shown similar beneficial effects in clinical trials [66,67]. However, some studies have found that Tramadol, Aripiprazole, and Fentanyl have not been associated with risk reduction in SREs. Thus, our results may provide support for further investigations. The model identified several medications that increased the risk of SREs. Such medications have also been reported to increase the risk of SREs in other studies [68]. Caution must be taken in interpreting the effect of medications on the prediction of SREs in that the model’s results do not account for drugs that may be indicators of comorbidities, e.g., sleep problems that may alter the risk of SREs.

The results of our study made it possible for clinicians to identify patients who have a higher risk of SREs and have additional insight of how to reduce this risk by identified risk factors. Clinicians will be able to adjust the medications to replace some drugs which increase the risk of SREs with drugs with the same class with less risk or focus on relieving the symptoms which may contribute most to the suicide risk. 

The ML-based random forest model provides a basis for clinicians to build similar models for different populations facing different disease risks. Our model is built with open source Python packages and trained based on EMR data. This means other researchers can test our model in other clinical samples. Also, our study can provide guidance for clinical institutions or other researchers to build their own models for other kinds of populations.

Unavoidably, there are limitations to this study. (a) the data was collected from hospitals affiliated with UPMC. External data for validation was not used and, if included, may have led to overfitting; (b) most clinicians prefer to treat diseases and disorders with particular combinations of drugs different from those used by other clinicians. This may cause bias in the results among institutions if such preferences are widely used in the hospital, despite alternative drug choices; (c) the high prediction performance of the model may due to the unique characteristics of the BDT patient subpopulation. The model may need further adjustment and optimization to apply it to other high-suicide risk populations or other disease states; (d) mis-diagnoses and biased prescriptions are two problems may cause errors in the predictions of SREs. PTSD and bipolar disorder may be mis-diagnosed as other diseases in their early stages, which may cause bias in our model, especially with younger patients. However, the ability to identify mis-diagnoses and biased prescriptions from the EMR is beyond the capability of our model; (e) though some medications, like lithium, may not be indicated for SREs, clinicians prescribe them for bipolar disorders to a greater extent due to its known anti-suicidal properties. This may be the situation in many clinical practices. 

The ML-based random forest model makes it possible for clinicians to identify subpopulations of patients who have a higher risk of SREs and to have additional insights to reducing this risk by identifying individual risk factors. Medications that increase the risk of SREs can be substituted with drugs having a lower risk or that focus on relieving symptoms that may contribute most to SREs. 

Using EMR information, a ML-based random forest model was constructed that predicts, with an accuracy of around 90%, if a patient will have an SRE within the following year of the diagnosis of both bipolar disorder and PSTD. The model extracts features that make contributions to the risk of SREs, which can be further utilized in mechanism studies. The model has great potential as a clinical tool that can aid clinicians in identifying high-risk individuals and to better guide patient clinical care. 

## Figures and Tables

**Table 1:** Baseline Patient Characteristics.

Characteristic	Suicide (Percentage)	Not Suicide (Percentage)	*p* Value *
	*N* = 205	*N* = 2963	
Gender			
Male	66 (32.2)	688 (23.2)	0.005
Female	139 (67.8)	2275 (76.8)	
Lithium Use			
Yes	16 (7.8)	221 (7.5)	0.964
Not	189 (92.2)	2742 (92.5)	
ED Visits			
10 ≤ X	15 (7.3)	93 (3.1)	0.003
5 ≤ X < 10	28 (13.7)	260 (8.8)	0.026
4	9 (4.4)	133 (4.5)	0.999
3	19 (9.3)	213 (7.2)	0.334
2	20 (9.8)	357 (12.0)	0.385
1	43 (21.0)	596 (20.1)	0.836
0	71 (34.6)	1311 (44.2)	0.009
Age			
Mean (SD)	35.06 (12.92)	38.45 (13.29)	<0.001

* *p* Values were generated with chi-square test.

**Table 2:** Model performance of all models *.

	K-Nearest Neighbors	Naïve Bayes	Decision Tree	Support Vector Machine	Logistic Regression	Random Forest
TP	182	200.6	146	114.2	111.8	171
FP	888	2732.6	103.8	1238.8	1074.6	17
TN	2075	230.4	2859.2	1724.2	1888.4	2946
FN	23	4.4	59	90.8	93.2	34
TPR	0.888	0.979	0.712	0.557	0.545	0.834
PPV	0.17	0.068	0.585	0.084	0.094	0.91
NPV	0.989	0.981	0.98	0.95	0.953	0.989

TP: True positive, TN: True negative, FP: False positive, FN: False negative, TPR: True positive rate or Sensitivity, PPV: Positive predictive value, NPV: Negative predictive value. * Values in the table are means from 5-fold stratified cross validation.

**Table 3:** Feature importance in random forest model.

Feature	Feature Importance
Age at both diagnosed	0.141
Disease category 5 in last year	0.081
Disease category 3 in last year	0.07
Disease category 1 in last year	0.061
Trazodone	0.055
Fentanyl	0.047
Disease category 11 in last year	0.039
Emergency department visits in last year	0.038
Lamotrigine	0.036
Sertraline	0.031
Disease category 6 in last year	0.031
Disease category 2 in last year	0.023
Quetiapine	0.023
Citalopram	0.022
Bupropion	0.021
Tramadol	0.021
Fluoxetine	0.018
Aripiprazole	0.017
Haloperidol	0.016
Venlafaxine	0.016
Lithium	0.015
Duloxetine	0.014
Buspirone	0.012
GENDER	0.012
Risperidone	0.011
Disease category 4 in last year	0.011
Mirtazapine	0.01
Ziprasidone	0.009
Olanzapine	0.009
Promethazine	0.008
Escitalopram	0.008
Amphetamine	0.006
Sumatriptan	0.006
Disease category 9 in last year	0.006
Amitriptyline	0.005
Chlorpromazine	0.005
Carbamazepine	0.005
Disease category 10 in last year	0.004
Paroxetine	0.004
Methadone	0.004
Disease category 12 in last year	0.003
Dextromethorphan	0.003
Lurasidone	0.003
Meperidine	0.003
Rizatriptan	0.003
Asenapine	0.002
Doxepin	0.002
Disease category 8 in last year	0.002
Vilazodone	0.001
Perphenazine	0.001
Nortriptyline	0.001
Thiothixene	0.001
Clomipramine	0.001
Ropinirole	0.001
Paliperidone	0.001
Eletriptan	0.001
Naratriptan	0
Zolmitriptan	0
Desvenlafaxine	0
Selegiline	0
Levomilnacipran	0
Milnacipran	0
Vortioxetine	0
Dihydroergotamine	0
Imipramine	0
Desipramine	0
Tapentadol	0
Clozapine	0
Fluphenazine	0
Disease category 7 in last year	0
Trifluoperazine	0
Almotriptan	0
Rasagiline	0
Brexpiprazole	0
Chlorpheniramine	0
Cariprazine	0
Fluvoxamine	0
Loxapine	0
Rotigotine	0
Dexmethylphenidate	0
Protriptyline	0
Tranylcypromine	0
Flibanserin	0
Amoxapine	0
Frovatriptan	0
Iloperidone	0
Maprotiline	0
Phenelzine	0
Pimozide	0
Nefazodone	0

**Table 4:** Performance of model retrained on selected features.

	TP	FP	TN	FN	TPR	PPV	NPV
Retrained model	171	14	2949	34	0.834	0.924	0.988

TP: True positive, TN: True negative, FP: False positive, FN: False negative, TPR: True positive rate or Sensitivity, PPV: Positive predictive value, NPV: Negative predictive value.

**Table 5:** Feature value distribution significance in positive and negative contributing groups.

Features	*T*	*P*	Percentage of 1 in Whole Population	Percentage of 1 with Positive Contribution	Percentage of 1 with Negative Contribution	FDR Adjusted *q* Value	Direction of Effect
Disease category 2 in last year	2065.444	<0.001	0.064	0.919	0.017	<0.001	SREs
Disease category 11 in last year	2239.822	<0.001	0.098	0.996	0.027	<0.001	SREs
Disease category 6 in last year	1750.073	<0.001	0.066	0.846	0.021	<0.001	SREs
Disease category 1 in last year	2193.804	<0.001	0.232	1	0.068	<0.001	SREs
Trazodone	1248.659	<0.001	0.17	0.996	0.101	<0.001	SREs
Sertraline	1205.388	<0.001	0.116	0	0.459	<0.001	No SREs
GENDER	681.776	<0.001	0.237	0.062	0.463	<0.001	No SREs
Haloperidol	489.011	<0.001	0.046	0.32	0.021	<0.001	SREs
Fentanyl	486.882	<0.001	0.188	0.002	0.317	<0.001	No SREs
Aripiprazole	428.686	<0.001	0.089	0.003	0.219	<0.001	No SREs
Lamotrigine	424.696	<0.001	0.146	0.001	0.264	<0.001	No SREs
Disease category 4 in last year	422.183	<0.001	0.034	0.261	0.014	<0.001	SREs
Ziprasidone	348.145	<0.001	0.037	0.202	0.013	<0.001	SREs
Risperidone	326.949	<0.001	0.063	0.378	0.043	<0.001	SREs
Mirtazapine	166.917	<0.001	0.063	0.186	0.037	<0.001	SREs
Quetiapine	127.63	<0.001	0.135	0.32	0.109	<0.001	SREs
Venlafaxine	119.404	<0.001	0.082	0.214	0.06	<0.001	SREs
Buspirone	111.025	<0.001	0.079	0.024	0.126	<0.001	No SREs
Disease category 5 in last year	108.425	<0.001	0.46	0.989	0.443	<0.001	SREs
Duloxetine	104.213	<0.001	0.069	0.175	0.048	<0.001	SREs
Tramadol	76.116	<0.001	0.131	0.038	0.162	<0.001	No SREs
Citalopram	63.62	<0.001	0.124	0.233	0.103	<0.001	SREs
Bupropion	18.005	<0.001	0.109	0.066	0.122	<0.001	No SREs
Fluoxetine	14.305	<0.001	0.092	0.065	0.107	<0.001	No SREs
Disease category 3 in last year	14.02	<0.001	0.749	0.907	0.744	<0.001	SREs
Promethazine	7.757	0.005	0.052	0.038	0.061	0.006	No SREs
Lithium	5.165	0.023	0.076	0.058	0.083	0.024	No SREs
Olanzapine	1.026	0.311	0.054	0.048	0.057	0.311	N/A

## Appendix A. ICD9 and ICD10 Codes for PTSD, Bipolar Disorder and Suicide Events

PTSD: 309.81, F43.10, F43.11, F43.12

Bipolar disorder: 296.8, 296.89, 296.7, F31.81, 296.5, F31.32, F31.89, 296.53, 296.54, F31.4, 296.6, 296.44, F31.75, 296, 296.52, F31.12, F31.30, F31.70, F31.2, F31.31, 296.4, F31.0, F31.62, F31.5, 296.64, 296.42, F31.76, F31.13, 296.62, F31.11, F31.60, 296.41, F31.61, 296.63, F31.77, F31.10, F31.71, 296.51, 296.46, 296.43, 296.55, F31.63, F31.73, F31.78, 296.45, 296.66, 296.61, F31.74, F31.64, 296.02, F31.72, 296.56, 296.01, 296.03, 296.65, 296.06, 296.6, 296.04, 296.8, 296.05

**Table A1:** Suicide-Related Events.

ICD Code	Diagnosis Name	#Patients in 205 Patients
R45.851	Suicidal ideations	83
V62.84	Suicidal ideation	78
E950.4	Suicide and self-inflicted poisoning by other specified drugs and medicinal substances	9
E950.3	Suicide and self-inflicted poisoning by tranquilizers and other psychotropic agents	7
E956	Suicide and self-inflicted injury by cutting and piercing instrument	6
E958.8	Suicide and self-inflicted injury by other specified means	5
T43.222A	Poisoning by selective serotonin reuptake inhibitors, intentional self-harm, initial encounter	2
X78.9XXA	Intentional self-harm by unspecified sharp object, initial encounter	2
E950.0	Suicide and self-inflicted poisoning by analgesics, antipyretics, and antirheumatics	1
E950.2	Suicide and self-inflicted poisoning by other sedatives and hypnotics	1
T14.91XA	Suicide attempt, initial encounter	1
T39.1X2A	Poisoning by 4-Aminophenol derivatives, intentional self-harm, initial encounter	1
T40.1X2A	Poisoning by heroin, intentional self-harm, initial encounter	1
T42.1X2A	Poisoning by iminostilbenes, intentional self-harm, initial encounter	1
T42.4X2A	Poisoning by benzodiazepines, intentional self-harm, initial encounter	1
T42.4X2D	Poisoning by benzodiazepines, intentional self-harm, subsequent encounter	0
T42.6X2A	Poisoning by other antiepileptic and sedative-hypnotic drugs, intentional self-harm, initial encounter	1
T43.212A	Poisoning by selective serotonin and norepinephrine reuptake inhibitors, intentional self-harm, initial encounter	1
T46.5X2A	Poisoning by other antihypertensive drugs, intentional self-harm, initial encounter	1
T50.902D	Poisoning by unspecified drugs, medicaments and biological substances, intentional self-harm, subsequent encounter	0
T65.92XD	Toxic effect of unspecified substance, intentional self-harm, subsequent encounter	0
T71.162A	Asphyxiation due to hanging, intentional self-harm, initial encounter	1
X78.8XXA	Intentional self-harm by other sharp object, initial encounter	1
X78.9XXD	Intentional self-harm by unspecified sharp object, subsequent encounter	0
X83.8XXA	Intentional self-harm by other specified means, initial encounter	1
E950.1	Suicide and self-inflicted poisoning by barbiturates	0
E950.5	Suicide and self-inflicted poisoning by unspecified drug or medicinal substance	0
E950.6	Suicide and self-inflicted poisoning by agricultural and horticultural chemical and pharmaceutical preparations other than plant foods and fertilizers	0
E950.7	Suicide and self-inflicted poisoning by corrosive and caustic substances	0
E950.9	Suicide and self-inflicted poisoning by other and unspecified solid and liquid substances	0
E951.0	Suicide and self-inflicted poisoning by gas distributed by pipeline	0
E951.8	Suicide and self-inflicted poisoning by other utility gas	0
E952.0	Suicide and self-inflicted poisoning by motor vehicle exhaust gas	0
E952.1	Suicide and self-inflicted poisoning by other carbon monoxide	0
E952.8	Suicide and self-inflicted poisoning by other specified gases and vapors	0
E953.0	Suicide and self-inflicted injury by hanging	0
E953.1	Suicide and self-inflicted injury by suffocation by plastic bag	0
E953.8	Suicide and self-inflicted injury by other specified means	0
E953.9	Suicide and self-inflicted injury by unspecified means	0
E954	Suicide and self-inflicted injury by submersion [drowning]	0
E955.0	Suicide and self-inflicted injury by handgun	0
E955.1	Suicide and self-inflicted injury by shotgun	0
E955.2	Suicide and self-inflicted injury by hunting rifle	0
E955.4	Suicide and self-inflicted injury by other and unspecified firearm	0
E955.9	Suicide and self-inflicted injury by firearms and explosives, unspecified	0
E957.0	Suicide and self-inflicted injuries by jumping from residential premises	0
E957.1	Suicide and self-inflicted injuries by jumping from other man-made structures	0
E957.9	Suicide and self-inflicted injuries by jumping from unspecified site	0
E958.0	Suicide and self-inflicted injury by jumping or lying before moving object	0
E958.1	Suicide and self-inflicted injury by burns, fire	0
E958.2	Suicide and self-inflicted injury by scald	0
E958.3	Suicide and self-inflicted injury by extremes of cold	0
E958.5	Suicide and self-inflicted injury by crashing of motor vehicle	0
E958.6	Suicide and self-inflicted injury by crashing of aircraft	0
E958.7	Suicide and self-inflicted injury by caustic substances, except poisoning	0
E958.9	Suicide and self-inflicted injury by unspecified means	0
T14.91	Suicide attempt	0
T14.91XD	Suicide attempt, subsequent encounter	0
T14.91XS	Suicide attempt, sequela	0
T36.0X2A	Poisoning by penicillins, intentional self-harm, initial encounter	0
T36.0X2D	Poisoning by penicillins, intentional self-harm, subsequent encounter	0
T36.1X2A	Poisoning by cephalosporins and other beta-lactam antibiotics, intentional self-harm, initial encounter	0
T36.3X2A	Poisoning by macrolides, intentional self-harm, initial encounter	0
T36.4X2A	Poisoning by tetracyclines, intentional self-harm, initial encounter	0
T36.8X2A	Poisoning by other systemic antibiotics, intentional self-harm, initial encounter	0
T37.5X2A	Poisoning by antiviral drugs, intentional self-harm, initial encounter	0
T37.8X2A	Poisoning by other specified systemic anti-infectives and antiparasitics, intentional self-harm, initial encounter	0
T38.1X2A	Poisoning by thyroid hormones and substitutes, intentional self-harm, initial encounter	0
T38.2X2A	Poisoning by antithyroid drugs, intentional self-harm, initial encounter	0
T38.3X2A	Poisoning by insulin and oral hypoglycemic [antidiabetic] drugs, intentional self-harm, initial encounter	0
T38.3X2D	Poisoning by insulin and oral hypoglycemic [antidiabetic] drugs, intentional self-harm, subsequent encounter	0
T38.5X2A	Poisoning by other estrogens and progestogens, intentional self-harm, initial encounter	0
T38.892A	Poisoning by other hormones and synthetic substitutes, intentional self-harm, initial encounter	0
T39.012A	Poisoning by aspirin, intentional self-harm, initial encounter	0
T39.012D	Poisoning by aspirin, intentional self-harm, subsequent encounter	0
T39.092A	Poisoning by salicylates, intentional self-harm, initial encounter	0
T39.1X2D	Poisoning by 4-Aminophenol derivatives, intentional self-harm, subsequent encounter	0
T39.312A	Poisoning by propionic acid derivatives, intentional self-harm, initial encounter	0
T39.312D	Poisoning by propionic acid derivatives, intentional self-harm, subsequent encounter	0
T39.392A	Poisoning by other nonsteroidal anti-inflammatory drugs [NSAID], intentional self-harm, initial encounter	0
T39.4X2A	Poisoning by antirheumatics, not elsewhere classified, intentional self-harm, initial encounter	0
T39.8X2A	Poisoning by other nonopioid analgesics and antipyretics, not elsewhere classified, intentional self-harm, initial encounter	0
T39.92XA	Poisoning by unspecified nonopioid analgesic, antipyretic and antirheumatic, intentional self-harm, initial encounter	0
T40.1X2D	Poisoning by heroin, intentional self-harm, subsequent encounter	0
T40.2X2A	Poisoning by other opioids, intentional self-harm, initial encounter	0
T40.2X2D	Poisoning by other opioids, intentional self-harm, subsequent encounter	0
T40.3X2A	Poisoning by methadone, intentional self-harm, initial encounter	0
T40.4X2A	Poisoning by other synthetic narcotics, intentional self-harm, initial encounter	0
T40.5X2A	Poisoning by cocaine, intentional self-harm, initial encounter	0
T40.5X2D	Poisoning by cocaine, intentional self-harm, subsequent encounter	0
T40.602A	Poisoning by unspecified narcotics, intentional self-harm, initial encounter	0
T40.602D	Poisoning by unspecified narcotics, intentional self-harm, subsequent encounter	0
T40.7X2A	Poisoning by cannabis (derivatives), intentional self-harm, initial encounter	0
T40.8X2A	Poisoning by lysergide [LSD], intentional self-harm, initial encounter	0
T40.8X2D	Poisoning by lysergide [LSD], intentional self-harm, subsequent encounter	0
T40.992A	Poisoning by other psychodysleptics [hallucinogens], intentional self-harm, initial encounter	0
T41.292A	Poisoning by other general anesthetics, intentional self-harm, initial encounter	0
T41.3X2A	Poisoning by local anesthetics, intentional self-harm, initial encounter	0
T42.0X2A	Poisoning by hydantoin derivatives, intentional self-harm, initial encounter	0
T42.3X2A	Poisoning by barbiturates, intentional self-harm, initial encounter	0
T42.4X2S	Poisoning by benzodiazepines, intentional self-harm, sequela	0
T42.5X2A	Poisoning by mixed antiepileptics, intentional self-harm, initial encounter	0
T42.6X2	Poisoning by other antiepileptic and sedative-hypnotic drugs, intentional self-harm	0
T42.6X2D	Poisoning by other antiepileptic and sedative-hypnotic drugs, intentional self-harm, subsequent encounter	0
T42.72XA	Poisoning by unspecified antiepileptic and sedative-hypnotic drugs, intentional self-harm, initial encounter	0
T42.8X2A	Poisoning by antiparkinsonism drugs and other central muscle-tone depressants, intentional self-harm, initial encounter	0
T43.012A	Poisoning by tricyclic antidepressants, intentional self-harm, initial encounter	0
T43.012D	Poisoning by tricyclic antidepressants, intentional self-harm, subsequent encounter	0
T43.022A	Poisoning by tetracyclic antidepressants, intentional self-harm, initial encounter	0
T43.022D	Poisoning by tetracyclic antidepressants, intentional self-harm, subsequent encounter	0
T43.1X2A	Poisoning by monoamine-oxidase-inhibitor antidepressants, intentional self-harm, initial encounter	0
T43.202A	Poisoning by unspecified antidepressants, intentional self-harm, initial encounter	0
T43.212D	Poisoning by selective serotonin and norepinephrine reuptake inhibitors, intentional self-harm, subsequent encounter	0
T43.222D	Poisoning by selective serotonin reuptake inhibitors, intentional self-harm, subsequent encounter	0
T43.292A	Poisoning by other antidepressants, intentional self-harm, initial encounter	0
T43.292D	Poisoning by other antidepressants, intentional self-harm, subsequent encounter	0
T43.3X2A	Poisoning by phenothiazine antipsychotics and neuroleptics, intentional self-harm, initial encounter	0
T43.3X2D	Poisoning by phenothiazine antipsychotics and neuroleptics, intentional self-harm, subsequent encounter	0
T43.4X2A	Poisoning by butyrophenone and thiothixene neuroleptics, intentional self-harm, initial encounter	0
T43.502A	Poisoning by unspecified antipsychotics and neuroleptics, intentional self-harm, initial encounter	0
T43.592A	Poisoning by other antipsychotics and neuroleptics, intentional self-harm, initial encounter	0
T43.592D	Poisoning by other antipsychotics and neuroleptics, intentional self-harm, subsequent encounter	0
T43.612A	Poisoning by caffeine, intentional self-harm, initial encounter	0
T43.622A	Poisoning by amphetamines, intentional self-harm, initial encounter	0
T43.622D	Poisoning by amphetamines, intentional self-harm, subsequent encounter	0
T43.632A	Poisoning by methylphenidate, intentional self-harm, initial encounter	0
T43.692A	Poisoning by other psychostimulants, intentional self-harm, initial encounter	0
T43.8X2A	Poisoning by other psychotropic drugs, intentional self-harm, initial encounter	0
T43.92XA	Poisoning by unspecified psychotropic drug, intentional self-harm, initial encounter	0
T44.1X2A	Poisoning by other parasympathomimetics [cholinergics], intentional self-harm, initial encounter	0
T44.3X2A	Poisoning by other parasympatholytics [anticholinergics and antimuscarinics] and spasmolytics, intentional self-harm, initial encounter	0
T44.4X2A	Poisoning by predominantly alpha-adrenoreceptor agonists, intentional self-harm, initial encounter	0
T44.6X2A	Poisoning by alpha-adrenoreceptor antagonists, intentional self-harm, initial encounter	0
T44.7X2A	Poisoning by beta-adrenoreceptor antagonists, intentional self-harm, initial encounter	0
T44.7X2D	Poisoning by beta-adrenoreceptor antagonists, intentional self-harm, subsequent encounter	0
T44.992A	Poisoning by other drug primarily affecting the autonomic nervous system, intentional self-harm, initial encounter	0
T45.0X2A	Poisoning by antiallergic and antiemetic drugs, intentional self-harm, initial encounter	0
T45.0X2D	Poisoning by antiallergic and antiemetic drugs, intentional self-harm, subsequent encounter	0
T45.2X2A	Poisoning by vitamins, intentional self-harm, initial encounter	0
T45.2X2D	Poisoning by vitamins, intentional self-harm, subsequent encounter	0
T45.4X2A	Poisoning by iron and its compounds, intentional self-harm, initial encounter	0
T45.512A	Poisoning by anticoagulants, intentional self-harm, initial encounter	0
T46.0X2A	Poisoning by cardiac-stimulant glycosides and drugs of similar action, intentional self-harm, initial encounter	0
T46.1X2A	Poisoning by calcium-channel blockers, intentional self-harm, initial encounter	0
T46.2X2A	Poisoning by other antidysrhythmic drugs, intentional self-harm, initial encounter	0
T46.3X2A	Poisoning by coronary vasodilators, intentional self-harm, initial encounter	0
T46.4X2A	Poisoning by angiotensin-converting-enzyme inhibitors, intentional self-harm, initial encounter	0
T46.4X2D	Poisoning by angiotensin-converting-enzyme inhibitors, intentional self-harm, subsequent encounter	0
T46.5X2D	Poisoning by other antihypertensive drugs, intentional self-harm, subsequent encounter	0
T46.6X2A	Poisoning by antihyperlipidemic and antiarteriosclerotic drugs, intentional self-harm, initial encounter	0
T46.7X2A	Poisoning by peripheral vasodilators, intentional self-harm, initial encounter	0
T46.8X2A	Poisoning by antivaricose drugs, including sclerosing agents, intentional self-harm, initial encounter	0
T47.0X2A	Poisoning by histamine H2-receptor blockers, intentional self-harm, initial encounter	0
T47.1X2A	Poisoning by other antacids and anti-gastric-secretion drugs, intentional self-harm, initial encounter	0
T47.4X2A	Poisoning by other laxatives, intentional self-harm, initial encounter	0
T47.6X2A	Poisoning by antidiarrheal drugs, intentional self-harm, initial encounter	0
T48.1X2A	Poisoning by skeletal muscle relaxants [neuromuscular blocking agents], intentional self-harm, initial encounter	0
T48.202A	Poisoning by unspecified drugs acting on muscles, intentional self-harm, initial encounter	0
T48.3X2A	Poisoning by antitussives, intentional self-harm, initial encounter	0
T48.3X2D	Poisoning by antitussives, intentional self-harm, subsequent encounter	0
T48.4X2A	Poisoning by expectorants, intentional self-harm, initial encounter	0
T48.5X2A	Poisoning by other anti-common-cold drugs, intentional self-harm, initial encounter	0
T48.6X2A	Poisoning by antiasthmatics, intentional self-harm, initial encounter	0
T49.0X2A	Poisoning by local antifungal, anti-infective and anti-inflammatory drugs, intentional self-harm, initial encounter	0
T49.6X2A	Poisoning by otorhinolaryngological drugs and preparations, intentional self-harm, initial encounter	0
T49.6X2D	Poisoning by otorhinolaryngological drugs and preparations, intentional self-harm, subsequent encounter	0
T50.2X2A	Poisoning by carbonic-anhydrase inhibitors, benzothiadiazides and other diuretics, intentional self-harm, initial encounter	0
T50.2X2D	Poisoning by carbonic-anhydrase inhibitors, benzothiadiazides and other diuretics, intentional self-harm, subsequent encounter	0
T50.3X2A	Poisoning by electrolytic, caloric and water-balance agents, intentional self-harm, initial encounter	0
T50.5X2A	Poisoning by appetite depressants, intentional self-harm, initial encounter	0
T50.6X2A	Poisoning by antidotes and chelating agents, intentional self-harm, initial encounter	0
T50.7X2A	Poisoning by analeptics and opioid receptor antagonists, intentional self-harm, initial encounter	0
T50.8X2A	Poisoning by diagnostic agents, intentional self-harm, initial encounter	0
T50.902A	Poisoning by unspecified drugs, medicaments and biological substances, intentional self-harm, initial encounter	0
T50.902S	Poisoning by unspecified drugs, medicaments and biological substances, intentional self-harm, sequela	0
T50.992A	Poisoning by other drugs, medicaments and biological substances, intentional self-harm, initial encounter	0
T50.992D	Poisoning by other drugs, medicaments and biological substances, intentional self-harm, subsequent encounter	0
T51.0X2A	Toxic effect of ethanol, intentional self-harm, initial encounter	0
T51.0X2D	Toxic effect of ethanol, intentional self-harm, subsequent encounter	0
T51.1X2A	Toxic effect of methanol, intentional self-harm, initial encounter	0
T51.2X2A	Toxic effect of 2-Propanol, intentional self-harm, initial encounter	0
T51.2X2D	Toxic effect of 2-Propanol, intentional self-harm, subsequent encounter	0
T51.2X2S	Toxic effect of 2-Propanol, intentional self-harm, sequela	0
T51.8X2A	Toxic effect of other alcohols, intentional self-harm, initial encounter	0
T51.92XA	Toxic effect of unspecified alcohol, intentional self-harm, initial encounter	0
T51.92XD	Toxic effect of unspecified alcohol, intentional self-harm, subsequent encounter	0
T52.0X2A	Toxic effect of petroleum products, intentional self-harm, initial encounter	0
T52.4X2A	Toxic effect of ketones, intentional self-harm, initial encounter	0
T52.8X2A	Toxic effect of other organic solvents, intentional self-harm, initial encounter	0
T54.0X2A	Toxic effect of phenol and phenol homologues, intentional self-harm, initial encounter	0
T54.1X2A	Toxic effect of other corrosive organic compounds, intentional self-harm, initial encounter	0
T54.2X2A	Toxic effect of corrosive acids and acid-like substances, intentional self-harm, initial encounter	0
T54.3X2A	Toxic effect of corrosive alkalis and alkali-like substances, intentional self-harm, initial encounter	0
T54.3X2D	Toxic effect of corrosive alkalis and alkali-like substances, intentional self-harm, subsequent encounter	0
T54.3X2S	Toxic effect of corrosive alkalis and alkali-like substances, intentional self-harm, sequela	0
T54.92XA	Toxic effect of unspecified corrosive substance, intentional self-harm, initial encounter	0
T54.92XS	Toxic effect of unspecified corrosive substance, intentional self-harm, sequela	0
T55.0X2A	Toxic effect of soaps, intentional self-harm, initial encounter	0
T55.1X2A	Toxic effect of detergents, intentional self-harm, initial encounter	0
T56.892A	Toxic effect of other metals, intentional self-harm, initial encounter	0
T56.892D	Toxic effect of other metals, intentional self-harm, subsequent encounter	0
T58.02XA	Toxic effect of carbon monoxide from motor vehicle exhaust, intentional self-harm, initial encounter	0
T58.92XA	Toxic effect of carbon monoxide from unspecified source, intentional self-harm, initial encounter	0
T59.892A	Toxic effect of other specified gases, fumes and vapors, intentional self-harm, initial encounter	0
T62.0X2A	Toxic effect of ingested mushrooms, intentional self-harm, initial encounter	0
T65.222D	Toxic effect of tobacco cigarettes, intentional self-harm, subsequent encounter	0
T65.222S	Toxic effect of tobacco cigarettes, intentional self-harm, sequela	0
T65.892A	Toxic effect of other specified substances, intentional self-harm, initial encounter	0
T65.892D	Toxic effect of other specified substances, intentional self-harm, subsequent encounter	0
T65.92XA	Toxic effect of unspecified substance, intentional self-harm, initial encounter	0
T65.92XS	Toxic effect of unspecified substance, intentional self-harm, sequela	0
T71.162D	Asphyxiation due to hanging, intentional self-harm, subsequent encounter	0
T71.192A	Asphyxiation due to mechanical threat to breathing due to other causes, intentional self-harm, initial encounter	0
X71.0XXS	Intentional self-harm by drowning and submersion while in bathtub, sequela	0
X71.3XXA	Intentional self-harm by drowning and submersion in natural water, initial encounter	0
X71.8XXA	Other intentional self-harm by drowning and submersion, initial encounter	0
X71.9XXA	Intentional self-harm by drowning and submersion, unspecified, initial encounter	0
X72.XXXA	Intentional self-harm by handgun discharge, initial encounter	0
X72.XXXD	Intentional self-harm by handgun discharge, subsequent encounter	0
X72.XXXS	Intentional self-harm by handgun discharge, sequela	0
X73.0XXA	Intentional self-harm by shotgun discharge, initial encounter	0
X74.01XA	Intentional self-harm by airgun, initial encounter	0
X74.8XXS	Intentional self-harm by other firearm discharge, sequela	0
X74.9XXA	Intentional self-harm by unspecified firearm discharge, initial encounter	0
X74.9XXD	Intentional self-harm by unspecified firearm discharge, subsequent encounter	0
X74.9XXS	Intentional self-harm by unspecified firearm discharge, sequela	0
X76.XXXA	Intentional self-harm by smoke, fire and flames, initial encounter	0
X76.XXXD	Intentional self-harm by smoke, fire and flames, subsequent encounter	0
X77.8XXA	Intentional self-harm by other hot objects, initial encounter	0
X78.0XXA	Intentional self-harm by sharp glass, initial encounter	0
X78.0XXD	Intentional self-harm by sharp glass, subsequent encounter	0
X78.1XXA	Intentional self-harm by knife, initial encounter	0
X78.1XXD	Intentional self-harm by knife, subsequent encounter	0
X78.8XXD	Intentional self-harm by other sharp object, subsequent encounter	0
X78.9XXS	Intentional self-harm by unspecified sharp object, sequela	0
X79.XXXA	Intentional self-harm by blunt object, initial encounter	0
X79.XXXD	Intentional self-harm by blunt object, subsequent encounter	0
X80.XXXA	Intentional self-harm by jumping from a high place, initial encounter	0
X80.XXXD	Intentional self-harm by jumping from a high place, subsequent encounter	0
X81.0XXA	Intentional self-harm by jumping or lying in front of motor vehicle, initial encounter	0
X81.8XXA	Intentional self-harm by jumping or lying in front of other moving object, initial encounter	0
X82.8XXA	Other intentional self-harm by crashing of motor vehicle, initial encounter	0
X83.2XXA	Intentional self-harm by exposure to extremes of cold, initial encounter	0
X83.8XXD	Intentional self-harm by other specified means, subsequent encounter	0
X83.8XXS	Intentional self-harm by other specified means, sequela	0

## Appendix B. Categories of Comorbid Diseases

Comorbid medical disorders were also documented and categorized into 12 disease categories that used only ICD9 codes [42].

Category 1 (ICD9: 291* or 292* or 303* or 304* or (305* and not 305.1))

Category 2 (ICD9: 295* or 301.2)

Category 3 (ICD9: 296* or 298.0 or 300.4 or 301.1 or 309* or 311*)

Category 4 (ICD9: 297* or (298* and not 298.0))

Category 5 (ICD9: 308* or (300* and not 300.4))

Category 6 (ICD9: 301* not 301.1 and not 301.2)

Category 7 (ICD9: 302*)

Category 8 (ICD9: 306* or 316*)

Category 9 (ICD9: 307*)

Category 10 (ICD9: 290* or 293* or 294* or 310*)

Category 11 (ICD9: 299* or 312* or 313* or 314* or 315*)

Category 12 (ICD9: 317* or 318* or 319*)

**Table A2:** Categories of Comorbid Diseases.

ICD9 Code	Disease Name	Category	ICD9 Code	Disease Name	Category
291	Alcohol-induced mental disorders	1	301 (not 301.1 or 301.2)	Personality disorders (not Affective personality disorder or Schizoid personality disorder)	6
292	Drug-induced mental disorders	1	302	Sexual and gender identity disorders	7
303	Alcohol dependence syndrome	1	306	Physiological malfunction arising from mental factors	8
304	Drug dependence	1	316	Psychic factor w oth dis.	8
305 (not 305.1)	Nondependent abuse of drugs (not Tobacco use disorder)	1	307	Special symptoms or syndromes not elsewhere classified	9
295	Schizophrenic disorders	2	290	Dementias	10
301.2	Schizoid personality disorder	2	293	Transient mental disorders due to conditions classified elsewhere	10
296	Episodic mood disorders	3	294	Persistent mental disorders due to conditions classified elsewhere	10
298	Depressive type psychosis	3	310	Specific nonpsychotic mental disorders due to brain damage	10
300.4	Dysthymic disorder	3	299	Autistic disorder-current	11
301.1	Affective personality disorder	3	312	Disturbance of conduct not elsewhere classified	11
309	Adjustment reaction	3	313	Disturbance of emotions specific to childhood and adolescence	11
311	Depressive disorder NEC	3	314	Hyperkinetic syndrome of childhood	11
297	Delusional disorders	4	315	Specific delays in development	11
298 (but not 2980)	Other nonorganic psychoses ( not Depressive type psychosis)	4	317	Mild intellectual disabilities	12
308	Acute reaction to stress	5	318	Other specified intellectual disabilities	12
300 (but not 300.4)	Anxiety, dissociative and somatoform disorders (not Dysthymic disorder)	5	319	Unspecified intellectual disabilities	12

## Appendix C. Distribution of All Categorical Features

**Table A3:** Distribution of all categorical features.

	*T*	*P*	Proportion of 1 in Whole Population	Proportion of 1 with Positive Contributions	Proportion of 1 with Negative Contributions	FDR Adjusted *Q* Value	Direction of Effect
Almotriptan	18.747	<0.001	0.013	0	1	<0.001	No SREs
Sertraline	1027.936	<0.001	0.117	0	0.41	<0.001	No SREs
Selegiline	250.75	<0.001	0.001	1	0	<0.001	SREs
Rotigotine	130.307	<0.001	0.009	0	1	<0.001	No SREs
Rizatriptan	136.995	<0.001	0.013	0.071	0.003	<0.001	SREs
Risperidone	32.548	<0.001	0.062	0.131	0.053	<0.001	SREs
Rasagiline	16.996	<0.001	0.014	0	1	<0.001	No SREs
Sumatriptan	355.971	<0.001	0.051	0.004	0.169	<0.001	No SREs
Quetiapine	34.748	<0.001	0.135	0.068	0.155	<0.001	No SREs
Promethazine	130.817	<0.001	0.053	0.008	0.1	<0.001	No SREs
Paroxetine	78.278	<0.001	0.032	0.008	0.065	<0.001	No SREs
Olanzapine	174.851	<0.001	0.055	0.184	0.032	<0.001	SREs
Disease Category 12 in last year	150.338	<0.001	0.011	0.098	0.004	<0.001	SREs
Mirtazapine	113.859	<0.001	0.063	0.013	0.106	<0.001	No SREs
Milnacipran	1014.193	<0.001	0.007	0	1	<0.001	No SREs
Protriptyline	120.749	<0.001	0.002	0	1	<0.001	No SREs
Tapentadol	67.865	<0.001	0.016	0	1	<0.001	No SREs
Thiothixene	27.267	<0.001	0.001	0.029	0	<0.001	SREs
Tramadol	855.263	<0.001	0.131	0.002	0.373	<0.001	No SREs
Disease Category 11 in last year	1879.785	<0.001	0.098	0.957	0.036	<0.001	SREs
Disease Category 9 in last year	95.725	<0.001	0.043	0.008	0.079	<0.001	No SREs
Disease Category 8 in last year	44.042	<0.001	0.005	0.042	0.002	<0.001	SREs
Disease Category 7 in last year	259.428	<0.001	0.012	0	1	<0.001	No SREs
Disease Category 6 in last year	2032.077	<0.001	0.066	0.979	0.022	<0.001	SREs
Disease Category 5 in last year	152.933	<0.001	0.459	0.992	0.436	<0.001	SREs
Disease Category 4 in last year	614.247	<0.001	0.034	0.364	0.014	<0.001	SREs
Disease Category 3 in last year	13.537	<0.001	0.749	0.906	0.743	<0.001	SREs
Disease Category 2 in last year	1610.239	<0.001	0.064	0.965	0.029	<0.001	SREs
Disease Category 1 in last year	1640.843	<0.001	0.234	1	0.11	<0.001	SREs
Ziprasidone	947.034	<0.001	0.037	0.51	0.014	<0.001	SREs
Vortioxetine	903.45	<0.001	0.008	0	1	<0.001	No SREs
Vilazodone	15.103	<0.001	0.006	0.021	0.004	<0.001	SREs
Trifluoperazine	549.894	<0.001	0.007	0	1	<0.001	No SREs
Trazodone	1068.226	<0.001	0.17	0.919	0.105	<0.001	SREs
Methadone	13.736	<0.001	0.024	0.04	0.017	<0.001	SREs
Meperidine	465.207	<0.001	0.022	0.22	0.006	<0.001	SREs
GENDER	1359.169	<0.001	0.237	0.033	0.624	<0.001	No SREs
Loxapine	422.51	<0.001	0.007	0	1	<0.001	No SREs
Amitriptyline	666.435	<0.001	0.047	0.002	0.259	<0.001	No SREs
Aripiprazole	1651.206	<0.001	0.088	0	0.573	<0.001	No SREs
Asenapine	162.998	<0.001	0.005	0.068	0	<0.001	SREs
Brexpiprazole	1268.74	<0.001	0.004	0	1	<0.001	No SREs
Bupropion	148.547	<0.001	0.109	0.013	0.158	<0.001	No SREs
Buspirone	169.298	<0.001	0.079	0.004	0.132	<0.001	No SREs
Cariprazine	283.31	<0.001	0.004	0	1	<0.001	No SREs
Chlorpheniramine	251.938	<0.001	0.008	0	1	<0.001	No SREs
Chlorpromazine	72.256	<0.001	0.011	0.054	0.005	<0.001	SREs
Clozapine	208.063	<0.001	0.019	0	1	<0.001	No SREs
Desipramine	92.504	<0.001	0.033	0	1	<0.001	No SREs
Desvenlafaxine	113.145	<0.001	0.007	0	0.09	<0.001	No SREs
Dihydroergotamine	1673.55	<0.001	0.008	0	1	<0.001	No SREs
Doxepin	15.421	<0.001	0.029	0.013	0.038	<0.001	No SREs
Dexmethylphenidate	481.108	<0.001	0.004	0	1	<0.001	No SREs
Fluvoxamine	1049.281	<0.001	0.006	0	1	<0.001	No SREs
Lithium	97.967	<0.001	0.075	0.01	0.109	<0.001	No SREs
Escitalopram	14.179	<0.001	0.049	0.024	0.058	<0.001	No SREs
Fentanyl	754.886	<0.001	0.188	0	0.385	<0.001	No SREs
Levomilnacipran	462.413	<0.001	0.009	0	1	<0.001	No SREs
Lamotrigine	341.173	<0.001	0.145	0	0.239	<0.001	No SREs
Flibanserin	21.497	<0.001	0.011	0	1	<0.001	No SREs
Imipramine	863.02	<0.001	0.008	0	1	<0.001	No SREs
Fluoxetine	39.147	<0.001	0.091	0.053	0.119	<0.001	No SREs
Fluphenazine	128.321	<0.001	0.016	0	1	<0.001	No SREs
Haloperidol	450.954	<0.001	0.047	0.32	0.023	<0.001	SREs
Naratriptan	11.737	0.001	0.004	0.019	0.002	0.001246	SREs
Amphetamine	9.941	0.002	0.031	0.018	0.039	0.002455	No SREs
Venlafaxine	8.86	0.003	0.082	0.048	0.089	0.003627	No SREs
Tranylcypromine	8.243	0.004	0.029	0	1	0.004765	No SREs
Disease Category 10 in last year	4.595	0.032	0.027	0.035	0.022	0.037565	SREs
Paliperidone	4.379	0.036	0.007	0.013	0.005	0.041657	SREs
Lurasidone	4.196	0.041	0.032	0.023	0.037	0.046775	No SREs
Zolmitriptan	3.292	0.07	0.002	0.006	0.001	0.07875	N/A
Ropinirole	2.34	0.126	0.016	0.022	0.013	0.139808	N/A
Duloxetine	2.079	0.149	0.069	0.084	0.066	0.162	N/A
Dextromethorphan	2.073	0.15	0.015	0.01	0.017	0.162	N/A
Citalopram	2.027	0.155	0.123	0.094	0.126	0.165197	N/A
Clomipramine	1.945	0.163	0.004	0.01	0.003	0.171468	N/A
Nortriptyline	1.71	0.191	0.012	0.016	0.01	0.198346	N/A
Perphenazine	1.127	0.288	0.017	0.013	0.019	0.295291	N/A
Carbamazepine	0.301	0.584	0.03	0.028	0.032	0.5913	N/A
Eletriptan	0.061	0.804	0.001	0.003	0.001	0.804	N/A
